# Pathological findings in rotation thromboelastometry associated with thromboembolic events in COVID-19 patients

**DOI:** 10.1186/s12959-021-00263-0

**Published:** 2021-02-11

**Authors:** Kristina Boss, Andreas Kribben, Bartosz Tyczynski

**Affiliations:** 1 Department of Nephrology, University Hospital Essen, University Duisburg-Essen, Hufelandstrasse 55, 45147 Essen, Germany; 2 Department of Medical Intensive Care I, University Hospital Essen, University Duisburg-Essen, Essen, Germany

**Keywords:** COVID-19, ROTEM®, Sepsis, Thromboelastometry, Thromboembolic event

## Abstract

**Background:**

Severe thromboembolic events are one of the major complications associated with COVID-19 infection, especially among critically ill patients. We analysed ROTEM measurements in COVID-19 patients with a severe disease course and in patients with severe sepsis.

**Methods:**

In this study, data obtained by extended analysis of haemostasis with standard laboratory tests and thromboelastometry of 20 patients with severe course of COVID-19 were retrospectively analysed and compared with similar data from 20 patients with severe sepsis but no COVID-19.

**Results:**

The thromboelastometry values obtained from 20 sepsis patients contained a maximum clot firmness above the normal range but among COVID-19 patients, hypercoagulability was much more pronounced, with significantly higher maximum clot firmness (FIBTEM: 38.4 ± 10.1 mm vs. 29.6 ± 10.8 mm; *P*  = 0.012; EXTEM: 70.4 ± 10.4 mm vs. 60.6 ± 14.8 mm; *P*  = 0.022). Additionally, fibrinogen levels were significantly higher among COVID-19 patients (757 ± 135 mg/dl vs. 498 ± 132 mg/dl, *P* < 0.0001). Furthermore, thromboelastometry showed fibrinolysis shutdown among COVID-19 patients with significantly lower maximum of lysis than among sepsis patients (EXTEM: 0.6 ± 1.2 % vs. 3.3 ± 3.7 %; *P*  = 0.013). Seven of 20 COVID-19 patients experienced thromboembolic events, whereas no patient in the sepsis group experienced such events.

**Conclusions:**

ROTEM analysis showed significantly different pathological findings characterized by hypercoagulability and fibrinolysis shutdown among COVID-19 patients with a severe disease course compared to patients with severe sepsis. These abnormalities seem to be associated with thromboembolic events.

## Introduction

The new coronavirus SARS-CoV-2, causing the current pandemic of COVID-19-disease, triggers severe pneumonia and pneumonitis that can eventually result in acute respiratory distress syndrome (ARDS). Patients with this condition experience a massive inflammatory reaction of the immune system, which can lead to an acute derangement of haemostasis [1]. COVID-19-associated coagulopathy is similar but not equal to the coagulopathy observed in sepsis induced coagulopathy (SIC) or disseminated intravascular coagulopathy (DIC). Additionally, the severity of coagulopathy is associated with the severity of COVID-19 [2].

It is known that several viruses can activate the coagulation system, e.g. HIV or Ebola virus and also during the treatment of COVID-19 patients thromboembolic events are one of the major complications [3, 4]. These thromboembolic events are based on several pathophysiologic mechanisms, including endothelial dysfunction, characterized by increased levels of von Willebrand factor; systemic inflammation, characterized by Toll-like receptor activation and a procoagulatory state, characterized by the activation of the tissue factor pathway [5]. Moreover, thrombosis can be triggered by hypoxia which develops in a number of COVID-19 patients, as well as an association with antiphospholipid antibodies has been observed in some cases [6–8]. On top of that, a cytokine storm which occurs often in critically ill patients, can interact crucially with mechanisms of coagulation and anticoagulation control. In the context of hyperinflammation, a serious malfunction of cytokine-controlled coagulation can occur, leading to disseminated intravascular coagulation (DIC) which is characterized by a massive intravascular activation of clotting and impaired physiological anticoagulation and fibrinolysis [9]. This form of deranged coagulation is also observed in sepsis [10, 11]. However, in critically ill COVID-19 patients an extreme hypercoagulable state rarely seen in regular DIC has been observed [12]. Among patients with SARS-CoV-2 infection, thromboembolic events seem to occur more frequently than among patients with other types of severe pneumonia and are sometimes not suspected before the patient’s death [13, 14].

Currently, different standard coagulation parameters are used to describe haemostasis disorders in COVID-19 patients. In contrast to standard tests for coagulation and fibrinolysis, such as activated partial thromboplastin time (aPTT) and the D-dimer level, which reflect only limited parts of the coagulation system, rotation thromboelastometry (ROTEM) analyses almost all steps of the coagulation process. Thromboelastometry allows the assessment of the strength, elasticity and dissolution of a blood clot. It takes into account inhibitors of clot formation and anticoagulant drugs. So, the involvement of both coagulation factors and platelets can be investigated [15, 16].

A direct comparison of these parameters between COVID-19 and sepsis patients has not yet been made. We analysed ROTEM measurements obtained from patients with severe COVID-19 and from patients with severe sepsis. Furthermore, we investigated possible associations between thromboelastometry results and thromboembolic events among COVID-19 patients.

## Materials and Methods

### Patients

 The study was conducted in a medical intensive care unit (ICU) at the University Hospital Essen, Germany, from March through June 2020. It was approved by the local ethics committee (20-9322-BO).

This study analysed data from a total of 40 patients; 20 of whom had tested positive for SARS-CoV-2 by nasopharyngeal swab and reverse transcription polymerase chain reaction assay. All 20 of the patients experienced a severe disease course and were treated in an ICU. Data from these patients were compared to data from 20 patients who met the criteria for severe sepsis but had no COVID-19 [17]. These patients were chosen for the best possible match with respect to age, sex, and disease severity. The sequential organ failure assessment (SOFA) score was calculated over the first 24 h after the patient met the criteria for severe sepsis [18]. SOFA-Score was applied for both study groups at the same point of time. Patients who received any anticoagulation treatment other than unfractionated heparin or who had heparin-induced thrombocytopenia were excluded from the study.

### Blood sampling and measurements

Standard laboratory values of coagulation measured in this study were aPTT, international normalized ratio (INR), D-dimer levels, and fibrinogen levels. For an extended evaluation of haemostasis, we performed ROTEM. Standard laboratory values were determined from routine blood samples with an additional sample of 3 ml trisodium citrate monovette solution for ROTEM analysis. All blood samples were analysed within 90 min after blood sampling and with the same instrument, in accordance with general practice [19].

Thromboelastometry analysis was performed with ROTEM® delta system (Tem Innovations, Munich, Germany) according to manufacturer´s instructions. The tests included four different measurements: EXTEM, INTEM, FIBTEM and APTEM, so both the involvement of coagulation factors and platelets could be investigated. Hyperfibrinolysis was measured by comparison of EXTEM and APTEM [20]. For each of the four tests 300 µl citrated whole blood was mixed with analysis solutions [21]. The parameters clotting time (CT), clot firmness 10 minutes after CT (A10), maximum clot firmness (MCF) and maximum lysis (ML) were collected. ROTEM analysis was performed for all investigated patients at the clinician´s request during ICU stay.

### Determination of thromboembolic events

Thromboembolic events were determined by computed tomography (CT) scan. All CT-scans of a patient during ICU stay were analysed. This included CT scans of the chest and of the abdomen with and without the use of contrast media, and cranial CT scans. CT scan was performed at clinician´s decision for both COVID-19 patients and sepsis patients without COVID-19.

### Statistical analysis

Data are presented as means and standard deviations (SD). Some datasets were excluded by the D’Agostino-Pearson test for normality. Therefore, the Mann-Whitney U tests were performed to determine statistical significance, which was set at the level of P < 0.05. Non-parametric data are presented as median and interquartile range (IQR). For all other datasets, unpaired t-tests were performed to determine statistical significance, which was set at the level of P < 0.05. GraphPad Prism 8 software was used for statistical analysis and graphical evaluation.

## Results

### Patient characteristics

A total of 40 patients were included in this study: 34 men (85 %) and 6 women (15 %). The average age was 62 years (range, 43–80 years). Gender and age distribution and disease severity were similar in both groups. The mean SOFA-Score at ICU admission was 11 for the COVID-19 group and 12 for the sepsis group. The most common types of sepsis among study patients were pneumosepsis (17 out of 20 patients (85 %) and sepsis due to blood stream infection (10 out of 20 patients (50 %)). All 40 patients required mechanical ventilation at ICU admission and still required mechanical ventilation during blood withdrawal.

In the COVID-19 group, more patients needed renal replacement therapy (75 % vs. 55 %). All patients received unfractionated heparin in at least a prophylactic dose as a continuous infusion. Detailed patient characteristics are shown in Table 1.

**Table 1:** Demographics and baseline characteristics

	COVID-19	Sepsis
**Baseline characteristics**		
Patients, n	20	20
Gender M/F, n	19/1	15/5
Age, y / range	62 (43–80)	62 (45–76)
**Comorbidities, n (%)**		
Hypertension	8 (40)	11 (55)
Diabetes	4 (20)	7 (35)
Cardiovascular disease	7 (35)	9 (45)
COPD	3 (15)	3 (15)
**Disease severity**		
Mechanical ventilation, n (%)	20 (100 %)	20 (100 %)
CVVHD, n (%)	15 (75)	11 (55)
SOFA-Score on ICU admission	11 ± 3	12 ± 3
Thromboembolic events, n (%)	7 (35)	0
Length of ICU stay, days (range)	14 (4–23)	11 (3–21)
Length of hospital stay, days (range)	23 (13–28)	27 (14–29)
ICU mortality, n (%)	9 (45)	8 (40)
Hospital mortality, n (%)	9 (45)	8 (40)

### Comparison of haemostasis parameters

The thromboelastometry values obtained from the 20 sepsis patients showed a broad distribution. MCF was above the normal range, as determined by FIBTEM (29.6 ± 10.8 mm). Additional signs of hypercoagulability were elevated fibrinogen levels and D-dimer levels.

Among COVID-19 patients, hypercoagulability was much more pronounced than among sepsis patients, as determined by MCF (FIBTEM: 38.4 ± 10.1 mm vs. 29.6 ± 10.8 mm; *P* = 0.012; EXTEM: 70.4 ± 10.4 mm vs. 60.6 ± 14.8 mm; *P* = 0.022). This substantially higher clot strength above standard values was observed in 18 of the 20 COVID-19 patients but in only 13 of the 20 septic patients. Additionally, fibrinogen level was significantly higher in the COVID-19 group (757 ± 135 mg/dl vs. 498 ± 132 mg/dl, *P* < 0.0001).

D-dimer levels were threefold higher among the COVID-19 patients than among the sepsis patients (*P* = 0.14). Furthermore, thromboelastometry showed fibrinolysis shutdown, with significantly lower ML among COVID-19 patients than among sepsis patients in EXTEM (0.6 ± 1.2 % vs. 3.3 ± 3.7 %; *P* = 0.013). Additionally, comparison of EXTEM and APTEM among COVID-19 patients showed no signs of hyperfibrinolysis. Detailed haemostasis information is shown in Table 2.

**Table 2:** Haemostasis parameters

	COVID-19	Sepsis	*P* value
**Standard laboratory values**			
Hb, g/dl (normal, 13.7–17.2)	9.5 ± 1	8.3 ± 1.5	
Plts, x 10^9^/l (normal, 140–320)	272 ± 152	140 ± 90	
INR	1.16 ± 0.16	1.20 ± 0.28	
aPTT, s (normal, 24.4–32.4) Heparin dose, IU/h, range (mean)	43 ± 12.9 400–1200 (730)	32 ± 7.6 400–1000 (480)	
Fibrinogen, mg/dl (normal, 188–384)	757 ± 135	498 ± 132	**< 0.0001**
D-dimer, mg/l (normal, < 0.52)	16.4 ± 10.7	5.4 ± 6.0	0.14
**EXTEM**			
*CT, s (normal, 38–79) Median 25 % percentile − 75 % percentile	90.5 76.2–104.5	81 67.3–93.3	0.112
*CFT, s (normal, 34–159) Median 25 % percentile − 75 % percentile	58 47.8–81.5	76 54.3–139.8	0.066
A10, mm (normal, 43–65)	64.6 ± 12.3	54.7 ± 16.4	0.076
MCF, mm (normal, 50–72)	70.4 ± 10.4	60.6 ± 14.8	**0.022**
ML % 60 (normal, 6 %-10 %)	0.6 ± 1.2	3.3 ± 3.7	**0.013**
**INTEM**			
*CT, s (normal, 100–240) Median 25 % percentile − 75 % percentile	293.5 220.8–429.5	232.5 188.3–248	**0.016**
*CFT, s (normal, 30–110) Median 25 % percentile − 75 % percentile	66.5 54–129.8	79 53.3–128.5	0.888
A10, mm (normal, 44–66)	62 ± 15	54.5 ± 16	0.158
MCF, mm (normal, 50–72)	68.6 ± 11.4	59.2 ± 15.8	0.057
**FIBTEM**			
MCF, mm (normal, 9–25)	38.4 ± 10.1	29.6 ± 10.8	**0.012**
**APTEM**			
*CFT, s (normal, 35–160) Median 25 % percentile − 75 % percentile	56 45.5–70.5	86.5 60.3–123	0.02
MCF, mm (normal, 53–72)	69.1 ± 11	56 ± 15.2	0.003

Asterixis marks non-parametric data with median and IQR instead of mean and SD

### Thromboembolic events

At least one CT scan (CT scan of the chest, the abdomen, cranial CT or combination of these) was performed in all patients analysed in this study. A CT scan of the chest was performed in all COVID-19 patients and in 13 out of 20 patients in the sepsis group. To rule out pulmonary embolism all CT-scans of the chest were carried out with contrast media. CT scans determined that severe ischemic events developed among 7 patients in the COVID-19 group despite anticoagulation with heparin. Three patients experienced pulmonary embolism, 1 experienced thrombosis of the splenic artery with splenic infarction, 1 experienced infarction and necrosis in both kidneys, and 1 experienced thrombosis of the right ulnar artery, radial artery, and brachial artery. The seventh patient experienced catheter-associated thrombosis of the jugular vein. Before these ischemic events were detected, all 7 of these patients had been treated with at least 800 IU/h of unfractionated heparin. The anticoagulation dose was determined by clinical decision of the attending physician. None of the patients had a history of thrombosis. In these seven patients with thromboembolic events, the ROTEM tests were performed between four days before and eleven days after onset of the ischemic events. There was no statistically significant difference in thromboelastometry results between COVID-19 patients with or without thromboembolic events. Among the seven patients with a thromboembolic event, ROTEM showed similar pathological findings, regardless of whether the analysis was performed before or after the detection of thromboembolic event. None of the patients met the criteria for sepsis-induced coagulopathy according to current scoring recommendations [22]. No thromboembolic events were detected among the sepsis patients.

## Discussion

### Key findings

Patients with COVID-19 were more likely to exhibit significant hypercoagulability and fibrinolysis shutdown as determined by thromboelastometry than were patients with severe sepsis. The pathological ROTEM pattern of COVID-19 patients differs significantly from that of sepsis patients. Furthermore, fibrinogen levels were significantly higher among COVID-19 patients.

### Comparison with previous studies

In this study, extended analysis of haemostasis by ROTEM detected hypercoagulability among COVID-19 patients with a severe disease course compared to patients with severe sepsis. Our results are in line with those of Spiezia et al., who found that MCF, as an indicator of hypercoagulability, was significantly higher among COVID-19 patients than among non-COVID-19 patients in an ICU [23].

Hypercoagulability is a frequent phenomenon among COVID-19 patients. A recent study by Panigada et al. found accelerated clot formation and increased clot stiffness, which was interpreted as consistent with hypercoagulability and severe inflammation rather than with DIC [24]. This type of coagulation disorder, with an activation of the tissue factor pathway and a remarkable consumption of coagulation factors, platelet activation, and fibrinolysis, is often found among patients with sepsis [23–25]. A severe course of COVID-19 may be complicated by DIC or SIC [26]. Tang et al. reported a mortality rate of 11.5 % among patients with COVID-19 pneumonia and noted that 71.4 % patients who did not survive exhibited an abnormal coagulation profile consistent with DIC [26].

To our knowledge, the present study is the first to compare haemostasis between COVID-19 patients and sepsis patients. Neither standard laboratory tests nor thromboelastometry showed a coagulation disorder compatible with DIC. The results were instead compatible with a hypercoagulability in the context of a severe inflammatory state, which was clearly more pronounced among COVID-19 patients than among sepsis patients, whose levels were already elevated.

A second key finding of this study is the occurrence of significant fibrinolysis shutdown among COVID-19 patients, defined as a maximal lysis in EXTEM after 60 minutes of mean 0.6 %. Fibrinolysis shutdown as a stand-alone aspect of deranged haemostasis in COVID-19 patients is a phenomenon that has recently come to the forefront of research and remains controversial. Wright et al. emphasized the high correlation between fibrinolysis shutdown and the occurrence of thromboembolic events and the need for renal replacement therapy, whereas Ibanez et al. did not confirm this finding [27, 28]. In a recent case series of three patients infusion of tissue plasminogen activator showed a beneficial effect in COVID-19 patients with ARDS [29]. This could hint to our finding of fibrinolysis shutdown as a central pathophysiological aspect. The divergence in observations regarding fibrinolysis shutdown may be due to the various levels of disease severity among the patient cohorts studied. We found that fibrinolysis shutdown occurs among critically ill patients with multiorgan failure. Its predictive value must be further investigated.

Moreover, patients with COVID-19 exhibited significantly higher D-dimer levels than the sepsis group. This aspect of the pathological coagulation situation is associated with the occurrence of ischemia as a serious complication among critically ill patients with COVID-19. It has been shown that 30-day mortality rates may be lower among patients treated with therapeutic anticoagulation, but the mortality rate among these cases is still high [30, 4]. A case series described by Zhang et al. found that 5 of 7 patients with acro-ischaemia died [31]. The authors reported that the clinical situation of the affected patients did not improve despite the administration of low-molecular-weight heparin.

We detected severe ischemic events in 7 of 20 (35 %) COVID-19 patients despite anticoagulation with heparin. All of them had exhibited elevated D-dimer levels, and 5 died during an ICU-stay. Although increased D-dimer levels are an unspecific sign of coagulation activation among patients with sepsis, our study found a more than threefold increase in D-dimer levels among COVID-19 patients. As previously described by Maatman et al., elevated D-dimer levels can predict venous thromboembolism in patients with severe COVID-19 [32]. That supports our finding of substantially increased D-dimer levels as one aspect of associations between coagulation parameters and thromboembolic events in COVID-19 patients.

The number of thromboembolic events among COVID-19 patients as reported in published studies is highly variable. For example, Klok et al. reported an incidence of pulmonary embolism in 81 % of patients, whereas Pavoni et al. reported thromboembolic events among only 5–30 % [33, 34]. This effect may be due to the small numbers of patients evaluated and the various degrees of disease severity in the studies [4]. We detected thromboembolic events with CT-scan, analogous to other studies. The detection of thromboembolic events by CT-scan might have a relevant bias aspect, e.g. if it is performed at clinician´s request. We performed more CT-scans in the COVID-19 group to rule out pulmonary embolism, which might have led to the detection of more thromboembolic events in this group. In the sepsis group not all patients (13 out of 20) had a CT-scan of the chest. Furthermore, the once performed CT-scan can only prove the existence of thrombosis or ischemia, but inadequately for how long it already exists.

We found no statistically significant differences in ROTEM analysis between COVID-19 patients with or without thromboembolic events. Nevertheless, it is possible that ROTEM analysis could be a possible predictor for thromboembolic events, but the study cohort of 7 vs. 13 patients is too small to fully address this aspect. However, thromboembolic events seem to be associated with these pathological ROTEM analyses, since no patient in the sepsis group experienced a thromboembolic event. Our findings of this association are supported by Mortus et al., who described a correlation between pathological thromboelastographic results and higher thromboses rates [35]. Moreover, the data of Creel-Bulos et al., showed a significantly higher rate of thromboembolic events in patients with fibrinolysis shut down in ROTEM analysis than in patients without fibrinolysis shutdown [36]. Therefore, in order to assess a predictive value of ROTEM measurements for the occurrence of thromboembolic events, sequential ROTEM measurements and corresponding CT-scans should be performed in a prospective study design.

### Strength and limitations

The present study has several strengths and limitations. To our knowledge, this is the first study to compare patients with COVID-19 and patients with severe sepsis in terms of the results of an extended haemostasis analysis performed with ROTEM thromboelastometry. No previous study has compared fibrinolysis shutdown in patients with COVID-19 patients and patients with sepsis. The results are limited by the monocentric retrospective study design and the small sample size and should be validated by additional studies with larger patient cohorts.

## Conclusions

ROTEM analysis showed significantly different pathological findings characterized by hypercoagulability and fibrinolysis shutdown among COVID-19 patients with a severe disease course compared to patients with severe sepsis. These abnormalities seem to be associated with thromboembolic events.

## Data Availability

All data are provided in the manuscript.

## Abbreviations

A10: Clot firmness 10 minutes after clotting time
aPTT: Activated partial thromboplastin time
ARDS: Acute respiratory distress syndrome
COPD: Chronic obstructive pulmonary disease
CT: Clotting time
CT-scan: Computer tomography scan
CFT: Clot formation time
CVVHD: Continuous veno-venous haemodiafiltration
DIC: Disseminated intravascular coagulation
Hb: Haemoglobin
ICU: Intensive care unit
INR: International normalized ratio
MCF: Maximum clot firmness
ML: Maximum lysis
Plts: Platelets
ROTEM: Rotation thromboelastometry
SOFA: Sequential organ failure assessment
